# Mixed-Methods Research in Diabetes Management via Mobile Health Technologies: A Scoping Review

**DOI:** 10.2196/diabetes.6667

**Published:** 2017-02-06

**Authors:** Cigdem Sahin, Patti-Jean Naylor

**Affiliations:** 1 Social Dimensions of Health Program University of Victoria Victoria, BC Canada; 2 School of Exercise Science, Physical and Health Education University of Victoria Victoria, BC Canada

**Keywords:** mHealth, self-management, methods, review

## Abstract

**Background:**

Considering the increasing incidence and prevalence of diabetes worldwide and the high level of patient involvement it requires, diabetes self-management is a serious issue. The use of mobile health (mHealth) in diabetes self-management has increased, but so far research has not provided sufficient information about the uses and effectiveness of mHealth-based interventions. Alternative study designs and more rigorous methodologies are needed. Mixed-methods designs may be particularly useful because both diabetes self-management and mHealth studies require integrating theoretical and methodological approaches.

**Objective:**

This scoping review aimed to examine the extent of the use of mixed-methods research in mHealth-based diabetes management studies. The methodological approaches used to conduct mixed-methods studies were analyzed, and implications for future research are provided.

**Methods:**

Guided by Arksey and O’Malley’s framework, this scoping review implemented a comprehensive search strategy including reviewing electronic databases, key journal searches, Web-based research and knowledge centers, websites, and handsearching reference lists of the studies. The studies focusing on mHealth technologies and diabetes management were included in the review if they were primary research papers published in academic journals and reported using a combination of qualitative and quantitative methods. The key data extracted from the reviewed studies include purpose of mixing, design type, stage of integration, methods of legitimation, and data collection techniques.

**Results:**

The final sample (N=14) included studies focused on the feasibility and usability of mHealth diabetes apps (n=7), behavioral measures related to the mHealth apps (n=6), and challenges of intervention delivery in the mHealth context (n=1). Reviewed studies used advanced forms of mixed-methods designs where integration occurred at multiple points and data were collected using multiple techniques. However, the majority of studies did not identify a specific mixed-methods design or use accepted terminology; nor did they justify using this approach.

**Conclusions:**

This review provided important insights into the use of mixed methods in studies focused on diabetes management via mHealth technologies. The prominent role of qualitative methods and tailored measures in diabetes self-management studies was confirmed, and the importance of using multiple techniques and approaches in this field was emphasized. This review suggests defining specific mixed-methods questions, using specific legitimation methods, and developing research designs that overcome sampling and other methodological problems in future studies.

## Introduction

### The Increasing Need for mHealth and Mixed-Methods Research in Diabetes Management

Increases in diabetes incidence and prevalence are a major concern in today’s health care system. There are nearly 385 million diabetic patients in the world, and almost 90% of them have type 2 diabetes, which can be treated with appropriate lifestyle and nutrition changes. Diabetes is a complicated disease and requires a high level of patient involvement; 95% of its management is patient initiated. Considering the additional 235 million patients with type 2 diabetes expected worldwide by the year 2035, the development of diabetic patients’ self-management skills is critical [1].

Mobile health (mHealth) apps that offer personalized, fast, cost-effective, and engaging services to patients have been used increasingly in diabetes self-management [2,3]. The “disease and treatment” apps category currently includes more than 2250 diabetes apps (iOS and Android systems in total). The number of mobile apps for diabetes is growing rapidly and market penetration is expected to grow to 7.8% by 2018, reaching 24 million diabetic patients [4].

Despite the increase in the diabetes cases and rapid growth in the mobile app market, research does not yet provide sufficient information about the factors fostering or hindering the adoption of these apps, patients’ attitudes toward using them, or their effectiveness in terms of health behavior change [2,5,6,7,8]. Therefore, the controversy over whether research methodologies or mHealth-based interventions are ineffective [9,10] still remains. Alternative study designs and more rigorous methodologies to advance mHealth research, especially in diabetes management, are strongly suggested [2,11].

There is a growing interest in using mixed-methods research in mHealth-based diabetes management studies because both diabetes self-management and mHealth studies require using different approaches, techniques, and measures cooperatively within an integrated perspective [7,12,13]. This scoping review examined the extent of mixed-methods research used in mHealth-based diabetes management studies. The methodological approaches used to conduct mixed-methods studies were also investigated, and implications for future mHealth and diabetes management research are provided.

### A Brief Review of Mixed-Methods Research

Mixed-methods research grew out of the “paradigm” controversy between positivist (quantitative) and constructivist (qualitative) research traditions in the 1980s and 1990s [14]. Mixed-methods research relies on both theory and practice to integrate knowledge from multiple approaches, perspectives, tools, positions, and opinions. The term “method” is used to cover a broad range of methodological (data collection techniques, design types, research methods, and so on) and related philosophical issues (eg, ontology, epistemology, axiology) [15].

The main goal of mixed-methods research is to use both quantitative and qualitative approaches to provide a better understanding of the research phenomena [16]. Because of the complementary strengths of different research paradigms, methodologies, and methods, mixed-methods research may provide new and different perspectives about an issue, expanding study findings beyond those produced by only one approach. Mixed-methods research increases the credibility of results when different approaches suggest the same conclusion, so it’s a value-added methodology [17,18].

Mixed-methods research is generally used for (1) triangulation of different approaches and methodologies to explain a single phenomenon; (2) complementarity of the research methods where one method is used to elaborate, illustrate, enhance, or clarify the results from another method; (3) sequential development of a study in which the results of one method are used to inform the other; (4) initiation of a study by using one method to find contradictions and paradoxes in findings from another method; and (5) expansion of a research study by using different methods to gain new perspectives or insights about a research problem [19].

Selecting an appropriate research design is a crucial step in a mixed-methods research. The purpose of a study and the nature of the research question help shape the design used in a mixed-methods study. In addition, using either concurrent or sequential time orientation affects study design, sampling, and data collection.

In a concurrent time orientation, data collection is completed for quantitative and qualitative phases of the study at approximately the same time to answer the same question. Both datasets are processed during data analysis and interpretation stages.

In a sequential time orientation, data can be obtained in stages, so the data from the first stage are used to shape the selection of data in the second stage (exploratory or explanatory sequential design). On the basis of their study purposes, questions, designs, and resources, researchers might place equal emphasis on both methods or use one of them primarily [16,20].

Integration is the central issue of mixed-methods research because it enables researchers to examine both types of data intensively [20]. Integration can be achieved at the method level or at the interpretation and reporting level. Bryman [21] reported that 57% of social science studies using mixed methods collected qualitative and quantitative data separately, while in approximately 27% both quantitative and qualitative data were derived from a single data source (eg, survey questionnaires including closed and open-ended questions). A growing scholarly interest has advanced mixed-methods research in various areas. Especially in health sciences, several important applications of mixed-methods research have been reported [22,23]. However, the majority of these studies failed to provide a detailed description of their data collection techniques, methods of analysis, stages of integration, and justifications for the use of mixed methods [24].

## Methods

The 5-stage scoping review framework developed by Arksey and O’Malley [25] was used to identify and examine the related literature in this review. This framework allows researchers to clearly describe the methods used at each stage to increase the transparency and replicability of the studies.

### Framework Stage 1: Identifying the Research Question

The research questions addressed in this review are as follows: (1) what is the extent and nature of mixed-methods research in mHealth-based diabetes management studies and (2) what are the current methodological approaches for designing and conducting mixed methods in these studies?

As a scoping review, this study did not intend to evaluate the scientific rigor of the selected studies as seen in systematic reviews [25]. It aimed to present the different ways in which researchers have used mixed methods.

### Framework Stage 2: Identifying Relevant Studies

Because the key purpose of the study was scoping the area of research, a comprehensive search strategy using multiple sources was used to identify all the relevant studies. Content-specific electronic databases, key journals, Web-based research and knowledge centers, websites, and handsearches of reference lists from studies and reviews were included in the search. The search was run between May 15, 2016, and June 30, 2016, and included all articles published by July 1, 2016. Table 1 presents the literature sources used for this review.

**Table 1:** Sources used to search the literature in this scoping review.

Source type	Literature sources used
Electronic databases	PubMed, CINAHL^a^, Web of Science, PsycINFO, Google Scholar
Key journal search	Journal of Medical Internet Research, The Journal of mHealth, The Diabetes Educator, The Journal of Mixed Methods Research, Journal of Diabetes, Journal of Diabetes Research, Journal of Telemedicine and Telecare, Telemedicine and e-Health
Research and knowledge networks	mHealth Evidence US NIDDK^b^ Diabetes Research Center, ResearchGate
Reference lists search	Systematic reviews, meta-analyses, narrative reviews, and similar article searches of the Web-based publishers

^a^CINAHL: Cumulative Index to Nursing and Allied Health Literature.

^b^NIDDK: National Institute of Diabetes and Digestive and Kidney Diseases.

Three sets of search term combinations were used in the search, and they were entered and combined using Boolean operators where applicable. Combination 1 was the most detailed and was used in each electronic database and on the mHealth Evidence website. It included “mobile health,” “mHealth,” “m-Health,” “mobile,” “mobile phone,” “smartphone,” “cellular phone,” “texting,” “text messaging,” “SMS,” “telemedicine,” “telehealth,” “telecare,” “telemonitoring,” “diabetes,” “diabetes mellitus,” “diabetic,” “mixed method,” “multi-method,” and “mix methodology.”

Because some studies do not explicitly indicate their methodologies as mixed methods, mix methodology, or multimethod, combination 2 was applied without using the methodology terms (mixed methods, multimethod, mix methodology). Because of the excessive number of records that resulted in the search with combination 2 in Google Scholar, PubMed, and Web of Science, this search was only completed in CINAHL (Cumulative Index to Nursing and Allied Health Literature) and PsycINFO databases.

Combination 3 used the terms most relevant to our research question. “Mobile health,” “diabetes,” and “mixed methods” search terms were used in US NIDDK (National Institute of Diabetes and Digestive and Kidney Diseases) Diabetes Research Center and in Google Scholar. Wide search options were used in terms of text availability, publication dates, and document formats, and only the English-language and academic journal filters were applied in electronic sources. ResearchGate was only used to search for the authors’ personal files and to obtain full-text articles. Table 2 summarizes the search term combinations used in each electronic source.

**Table 2:** Search combinations used in electronic sources.

Search terms	Web of Science	CINAHL^a^	PubMed	PsycINFO	Google Scholar	mHealth Evidence	US NIDDK^b^ Diabetes Research Center
Combination 1	✓	✓	✓	✓	✓	✓	
Combination 2	✓	✓	✓	✓	✓		
Combination 3					✓		✓

^a^CINAHL: Cumulative Index to Nursing and Allied Health Literature.

^b^NIDDK: National Institute of Diabetes and Digestive and Kidney Diseases.

### Framework Stage 3: Study Selection

Several inclusion and exclusion criteria were applied to the identified studies. The review included the primary research studies published in English-language academic journals that focused on using mHealth technologies in the context of type 1, type 2, or gestational diabetes management and included a combination of qualitative and quantitative methods.

**Figure 1:**
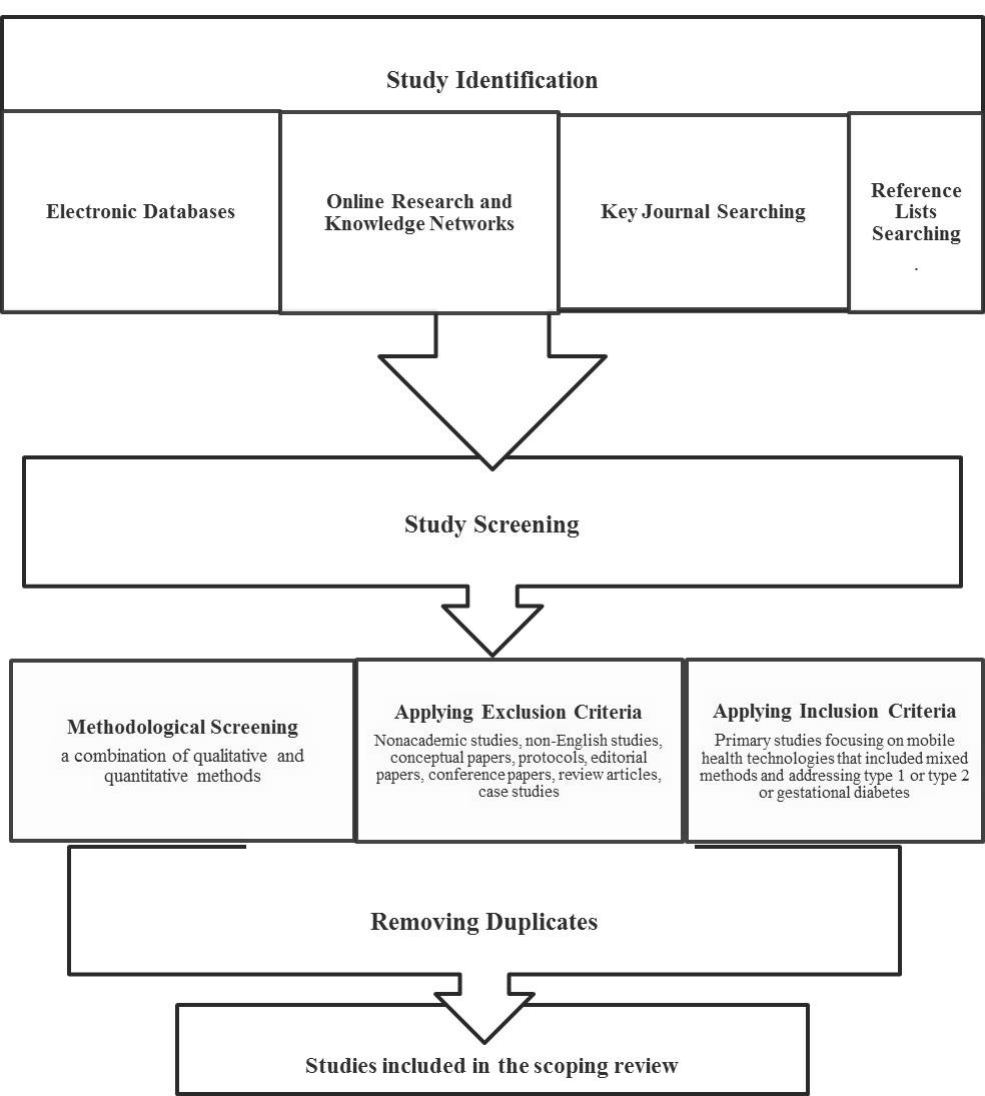
Study selection process.

In this review, the term “mobile health technologies” was used to cover mobile phones, portable monitoring devices (ie, accelerometer), and wireless devices used in medical care (ie, cell phones). Studies whose focus was Internet (computer)-based, telephone (landline)-based, or home-based monitoring were excluded. Examples of such studies include a mixed-methods diabetes telemonitoring study [26], using a Web-enabled glucometer for self-monitoring blood glucose, and a home-based monitoring study [27] including a randomized controlled trial and a series of interviews that used a transmission device to be attached to an analog telephone line or via USB to a computer to upload blood glucose and blood pressure measurements to a server. In addition, there were telephone coaching and counseling studies (ie, [28,29]) that did not match the inclusion criteria. The studies related to diabetic retinography were also excluded. Figure 1 presents the article selection process in this review.

### Framework Stage 4: Charting the Data

The descriptive-analytical method [25] was followed in order to standardize and chart key items of information acquired from the reviewed articles. The information collected and charted included the following elements:

- Authors, year of publication
- Main purpose of the study
- Recognition of mixed methods (mixed-methods terminology used or not)
- Purpose of mixing
- Formal mixed-methods research question formulated (yes or no)
- Prioritized method
- Design type
- Stage of integration
- Legitimation methods used
- Sources of qualitative data
- Sources of quantitative data
- Limitations described

Tables 3-5 present the definitions used to analyze the design types (Table 3), integration strategies (Table 4), and legitimation methods (Table 5) in this review.

**Table 3:** Common design types used in mixed-methods research.

Designs^a^		Objectives
Concurrent		This design aims to compare and contrast the results of both quantitative and qualitative findings, or to validate or expand quantitative results with qualitative data. This design is also labeled as parallel or convergent design. The researchers use different methods complementarily to investigate the same topic.
**Sequential**		
	Exploratory	If a study has one dataset built on the results from the other, it is classified as a sequential design. It is a 2-phase mixed-methods design that collects only one type of data at a time. This design aims to explore first by placing a qualitative phase before a quantitative phase, and the data from the first phase are used to develop the second phase.
	Explanatory	This design aims to clarify or interpret unexpected or confusing results from the quantitative phase with a follow-up qualitative phase. It can also be used to form groups based on quantitative results and monitor the groups through follow-up qualitative research.
Embedded		This includes the combination of both quantitative and qualitative data, but one data type has a supportive, secondary role within the overall design. One type of data is embedded within a methodology adapted by the other data type. It can be either a 1- or a 2-phase study. Unlike the conventional mixed-methods researchers, who think both methods should answer the same question in the research, some researchers have different questions requiring different types of data. Some complex interventions and experimental studies need embedded designs because this design is more manageable in terms of time and resources.
Multiphase		This design includes multiple qualitative, quantitative, or mixed measurement phases conducted over time and linked together so that one phase builds on another with a common overall objective. They usually include convergent and sequential elements.

^a^Developed based on the frameworks presented in Creswell [20], Creswell and Plano Clark [30], and Creswell et al [22].

**Table 4:** Integration through methods.

Approach^a^	Description
Connecting	One dataset links to the other through sampling.
Building	One dataset informs the data collection approach of the other.
Merging	The two datasets are brought together for analysis.
Multiple integration	Data collection and analysis are linked at multiple points.

^a^Adapted from Fetters et al [31].

**Table 5:** Methods of legitimation.

Approach^a^	Description
Inside-outside legitimation	Insider (emic) viewpoint refers to viewpoint of a group member while outsider (etic) viewpoint refers to an objective viewpoint gathered from an external source. Peer reviews, expert reviews, participant views, and team members’ views are used for legitimation.
Paradigmatic or philosophical validity	Successful integration of philosophical and methodological beliefs, defining the paradigm assumptions explicitly, and conducting research accordingly are the main indicators of legitimation.
Commensurability legitimation	This type of validity is obtained when researchers develop a third, mixed view, which helps them make broader and richer explanations about their study conclusions.
Weakness minimization	This type of validity is related to the integration of the research; researchers must continuously work to have nonoverlapping weaknesses while planning and designing their study.
Sequential legitimation	This type of validity is used to understand whether the sequential order of qualitative and quantitative phases in a study influences the results.
Conversion legitimation	By quantifying the narrative descriptions and creating a narrative profile for quantitative results (qualitizing), researchers can interpret their data in a broader perspective.
Sample integration	This type of validity refers to making appropriate generalizations from mixed samples. The relationship between the sampling designs in quantitative and qualitative phases is an important indicator of validity.
Sociopolitical legitimation	In order to achieve this type of legitimation, mixed-methods researchers should advocate pluralism of perspectives and try to build a practical theory or result that research consumers will find valuable.
Multiple legitimation	This type of validity indicates the extent to which all the pertinent validities (quantitative, qualitative, and mixed) are addressed and resolved successfully.

^a^Adapted from Onwuegbuzie and Johnson [32].

## Results

### Framework Stage 5: Collating, Summarizing, and Reporting the Results

#### Collating and Summarizing the Results

The database search revealed 8 articles; with handsearches of reference lists and key journals, a total of 14 articles were identified. The final sample included studies focusing on feasibility and usability of mHealth diabetes technologies (n=7), behavioral measures related to mHealth apps (n=6), and challenges in patient recruitment, fidelity, and intervention delivery in the context of mHealth (n=1).

For this scoping review, the first author derived the data from the articles and completed the initial coding, which was verified by the second author. In addition, an independent researcher (an experienced researcher and doctoral student in social sciences) separately coded the articles based on the study design framework used in this review. Comparing the results revealed an interrater reliability level of more than 90%.

The characteristics of mixed-methods research used in the reviewed studies and the summary of the findings are presented in Tables 6 and 7. Table 6 presents the data gathered on formal recognition of mixed methods and purpose of using mixed methodology, indicating a formal mixed-methods question, priority of the methods used, stages of integration, and the design type used in the studies. Table 7 lists the data gathered on legitimation methods, qualitative and quantitative sources of data, and limitations as described in the studies.

**Table 6:** The characteristics of mixed-methods research in mHealth-based diabetes management studies examined in this review (part 1).

Author, year	Main purpose of the study	Recognition of MM^a^	Purpose of mixing	Formal MM research question	Prioritized method	Stage of integration	Design type
Allen et al, 2009 [33]	To assess feasibility and acceptability of continuous glucose monitoring and accelerometer technology in exercising type 2 diabetic patients	Yes, as multimethod	Complementarity	No	Equal	Sampling and interpretation	Explanatory sequential
Baron et al, 2015 [34]	To identify the challenges related to recruitment, fidelity, implementation, and context of mobile telehealth interventions targeting diabetic patients	Yes	Complementarity: different measures for different parts of the research phenomenon	No	QUAN^b^	Sampling, data collection, data analysis, and interpretation	Embedded
Baron et al, 2016 [35]	To examine the behavioral effects of a mobile phone–based home telehealth intervention in diabetic patients	No	Triangulation	No	QUAN	Sampling and interpretation	Embedded
Burner et al, 2013 [36]	To explore the attitudes of inner-city Latino patients toward TExT-MED^d^ program and other health information sources	Yes	Initiation: the qualitative study was conducted to understand the contradictory findings of the quantitative method	No	Equal	Sampling and interpretation	Explanatory sequential
Carroll et al, 2007 [37]	To evaluate user satisfaction with an mHealth diabetes monitoring system	Yes	Sequential development	No	QUAN	Discussion or interpretation	Exploratory sequential
Franklin et al, 2008 [38]	To explore the interactions of type 1 patients with SMS text messaging support	Yes	Triangulation	No	Equal	Sampling, data analysis, and interpretation	Concurrent
Froisland et al, 2012 [39]	To explore ways mobile apps can be used to monitor adolescents with type 1 diabetes	Yes	Triangulation	No	QUAL^c^	Sampling, data collection, and interpretation	Embedded
Georgsson and Staggers, 2016 [40]	To test the feasibility of a multimethod approach for patients’ experienced usability of a diabetes mHealth system	Yes, as multimethod	Triangulation	No	Equal	Sampling, data analyses, and interpretation	Concurrent
Grindrod et al, 2014 [41]	To examine the usability and usefulness of mobile medication apps with older adults	Yes	Triangulation	No	Equal	Sampling, interpretation, and data analysis	Concurrent
Jones et al, 2015 [42]	To evaluate the attitudes of American Indian women toward postpartum intervention approaches (including mHealth) and risk factors for developing gestational diabetes	Yes	Complementarity: different measures for different parts of the research phenomenon	No	QUAL	Sampling and interpretation	Embedded
Nundy et al, 2014 [43]	To investigate the behavioral effects of a theory-driven mobile phone–based intervention using an automated, interactive SMS text messaging system	Yes	Triangulation	No	QUAN	Sampling and interpretation	Embedded design
Osborn and Mulvaney, 2013 [44]	To examine the capability of an SMS text messaging and interactive voice response intervention for low-income adults with type 2 diabetes mellitus	Yes	Sequential development	No	Equal	Sampling, data analysis, and interpretation	Embedded
Verwey et al, 2016 [45]	To examine the reach, implementation, and satisfaction with a counseling tool combining an accelerometer, a mobile phone, and a Web application	Yes	Triangulation	No	Equal	Sampling, interpretation, and data analysis	Embedded
van der Weegen et al, 2014 [46]	To test the usability of a monitoring and feedback tool targeting diabetic patients	Yes	Sequential development	No	Equal	Sampling, data collection, data analysis, and interpretation	Multiphase study

^a^MM: mixed methods.

^b^QUAN: quantitative.

^c^QUAL: qualitative.

^d^TExT-MED: Trial to Examine Text Message–Based mHealth in Emergency Department Patients With Diabetes.

**Table 7:** The characteristics of mixed-methods research in mHealth-based diabetes management studies examined in this review (part 2).

Author, year	Legitimation methods described	Sources of qualitative data	Sources of quantitative data	Limitations described
Allen et al, 2009 [33]	Inside-outside legitimation	A 1-hour, structured, focus group interview (n=7) following the completion of the quantitative phase. Field notes were also taken on key discussion points and observations (eg, body language and group mood).	Descriptive measures of the sample (n=9). Wearable Continuous Glucose Monitoring System, activity monitor data, and activity counts reviewed for each participant.	Small sample size and lack of control group setting in quantitative phase were reported.
Baron et al, 2015 [34]	Inside-outside legitimation	Interviews, meetings, field notes, and communications between team members.	A 9-month randomized controlled trial (n=81) to assess intervention delivery and fidelity (patients and nurses).	A possible sample selection bias was indicated.
Baron et al, 2016 [35]	Inside-outside legitimation and conversion legitimation (quantizing the qualitative data)	Semistructured interviews (n=26) on perceived effects of mobile telehealth system on diabetes self-management.	A randomized controlled trial (n=81) with intervention and control groups was conducted. Self-report measures of self-efficacy, illness beliefs, and self-care were taken at baseline and 3- and 9-month points.	Sample size was indicated as insufficient to make generalizations.
Burner et al, 2013 [36]	Inside-outside legitimation	Two focus groups of 90-minute duration, one in English, one in Spanish (n=8), were conducted with a structured guide.	A 1-month bilingual diabetes SMS text messaging intervention (n=23). Quantitative data included demographic, clinical, and biometric data of patients, and measures of health behaviors, knowledge, and beliefs were taken.	Small sample size was indicated as a limitation to the generalizability of the results.
Carroll et al, 2007 [37]	Weakness minimization legitimation: large focus groups to support small-scale usability test	A series of focus groups (10; n=59) was conducted before testing a prototype cell phone with a glucose monitoring system.	A pilot usability test to evaluate satisfaction with the new system (n=10). A 15-item questionnaire is used.	Sample size and sample selection, intervention duration, and incentives to the participants were seen as barriers to generalizability of findings.
Franklin et al, 2008 [38]	Inside-outside legitimation and conversion legitimation	Content analysis of text messages and messaging patterns of a 12-month Sweet Talk intervention period.	A 12-month 3-armed randomized controlled trial of a text messaging support system, Sweet Talk (n=64), was conducted. Observational data on messaging patterns were triangulated with patient clinical and demographic data. Post hoc analyses combining qualitative data and demographic variables were made.	Small sample size was indicated as a limitation to generalizability of the results.
Froisland et al, 2012 [39]	Inside-outside legitimation	Semistructured in-depth interviews lasting between 45 and 90 minutes (n=12) were conducted at the end of the quantitative phase.	A pilot test of 2 mobile apps (n=12), after a 3-month trial.	Possible sampling bias, small sample size, and short intervention period were indicated.
Georgsson and Staggers, 2016 [40]	Inside-outside legitimation, conversion legitimation	Think aloud protocol and open-ended interviews (15-20 minutes) were conducted (n=20).	First, a brief demographic questionnaire, and, at the end of the intervention, a posttest questionnaire measuring the usability of an interactive SMS^a^- text messaging system for a randomly selected sample of patients with diabetes (n=10) were conducted.	Using a convenient sample frame and the novelty of the system to patients were indicated as a limitation to the generalizability of the findings.
Grindrod et al, 2014 [41]	Outside legitimation, sequential legitimation, sample integration legitimation	A 10-minute group discussion of what medication management meant before each usability evaluation and 30-minute focus group discussion after each session (n=35).	A 2-hour usability testing (n=35) of different mobile apps using a 10-item system usability scale and a visual analog scale was used.	Short intervention period was indicated as a limitation of the study.
Jones et al, 2015 [42]	Inside legitimation	Four focus groups consisting of 2-5 participants (n=11 in total) were conducted, maximum duration of 60 minutes. Individual interviews (n=15) ranged from 25-45 minutes.	A cross-sectional study (n=26) was conducted with eligible group of patients. The questionnaire included measures for personal and family health history and technology feasibility and acceptability.	The purposive sampling and small sample size were seen as barriers to the generalizability of the results.
Nundy et al, 2014 [43]	Inside-outside legitimation	Approximately 1-hour, semistructured, in-depth interviews (n=14) based on topic guides and open-ended questions after the intervention.	A longitudinal observational cohort study (n=74) was conducted and data were collected at baseline, 3 months (mid-intervention), and 6 months (end of intervention). Diabetes self-care, medication adherence, self-efficacy, health beliefs, and social support measures were used.	The limitations were described as the lack of control group measure and sole use of SMS text messaging intervention, which may create a causality problem. Sample size was small to make proper generalizations.
Osborn and Mulvaney, 2013 [44]	Inside-outside legitimation	Motivational interviewing, face-to-face interviews (n=20) before and after trial, at baseline, and after week 3.	Secondary research: previous descriptive data on target population obtained, and self-administered daily text messages and interactive voice response calls are collected for analysis (n=20).	Sampling size was indicated as small to generalize results.
Verwey et al, 2016 [45]	Inside and outside legitimation, conversion legitimation, sample integration	30-Minute semistructured telephone interviews with the nurses about the receipt of intervention and the evaluation forms regarding consultations were used.	A longitudinal 3-armed cluster randomized controlled trial in a total of 24 family practice locations; evaluation questionnaire after intervention with practice nurses (n=20) and patients (n=131; 71 with type 2 diabetes and 42 with chronic obstructive pulmonary disease).	A possible sample bias was indicated as a limitation of the study.
van der Weegen et al, 2014 [46]	Multiple legitimation: weakness minimization, conversion legitimation, inside-outside legitimation	Heuristic evaluation with 6 experts, thinking aloud procedure and video recordings of 5 patients at two different stages, a series of interviews with patients in the pilot test.	A usability test with 5 patients, a pilot test in real-life settings with 20 patients, and a poststudy system usability test were conducted.	The small sample size was indicated as a limitation to the generalizability of the findings.

^a^SMS: short message service.

#### Reporting the Results

##### Recognizing Mixed Methods and Purpose of Using Mixed Methods

According to Creswell and Plano Clark [47], mixed-methods studies should have a properly defined methodology and a formal terminology. This review shows that almost all the studies (13/14, 93%) defined their methodologies explicitly as either mixed methods or multimethod.

Only a few studies (4/14, 29%; Verwey et al [45], Georgsson and Staggers [40], Froisland et al [39], and Franklin et al [38]) used mixed-methods terminology or explained its purpose explicitly. For example, Franklin et al [38] stated that their purpose was to triangulate the messaging patterns and contents of an automated, scheduled SMS text messaging with diabetic patients’ clinical and demographic data, and Froisland et al [39] reported that they triangulated the usability assessments of a picture-based diabetes diary app and an SMS (short message service) text messaging with semistructured, in-depth interviews and field notes.

In other studies, information about the purpose of mixing methods had to be extracted from the methodology and discussion sections of the studies. Particularly for the embedded design studies, a decision rule was used based on the phases of a study because they could signify the purpose of mixing the methods in a study [30]. If different measurements using different types of data were addressing different parts of the research phenomena, it was evaluated as complementarity [30]. For example, in an embedded design study, the attitudes of a group of American Indian women toward potential Internet or mHealth interventions were examined using qualitative interviews and focus groups that were complementary to a cross-sectional study assessing their risk perceptions of diabetes [42]. If one method was embedded into the other method and used to compare and validate its results, it was evaluated as triangulation. For example, Nundy et al [43] conducted a longitudinal cohort study and compared and validated the results with in-depth interviews. In addition, if one type of data was used to inform the other type during the sequential development of a study, its mixing purpose was evaluated as sequential development. For example, motivational interviews before the intervention and follow-up phone interviews during the testing period were used in a study focusing on the development and feasibility of a text messaging and interactive voice response intervention [44].

In total, the purpose of mixing methods was triangulation in 7 studies [35,38,39,40,41,43,45], complementarity in 3 studies [33,34,42], sequential development in 3 studies [37,44,46], and initiation in 1 study [36].

##### Design Types

Because the studies did not use a formal mixed-methods terminology when describing their design types, stages of integration, and priority of the methods they used, information on these categories was extracted from the methodology sections of the studies.

The most common design used by the studies was embedded design (n=7). These studies (ie, [34,39,42,43,45]) were designed to mix different datasets at the design level. For example, Vervey et al [45] conducted a 3-armed cluster randomized controlled trial with nurses and patients to evaluate the physical activity counseling process with and without the use of mobile technology. During the intervention, data were gathered from nurses who interviewed the patients periodically, collected evaluation forms, and logged data. They conducted in-depth interviews with nurses after the trial to compare and cross-validate their findings.

There were 3 concurrent design studies in the review [38,40,41]. In their text messaging intervention, Franklin et al [38] conducted a content analysis and derived qualitative themes from the messaging patterns, which were also analyzed by quantitative methods. In addition, 3 sequential design studies were identified: 1 exploratory [37] and 2 explanatory design studies [33,36]. After completing the qualitative phase and establishing the framework of the research, Carroll et al [37] implemented a usability test of a diabetes monitoring system. On the contrary, Burner et al [36] conducted a focus group study to clarify the results of their quantitative study, measuring behavioral effects of an mHealth intervention in Latino diabetic patients.

The only multiphase design study in this review was the study by van der Weegen et al [46], which measured the usability of a monitoring tool targeting diabetic patients. They conducted different qualitative and quantitative measurements focusing on different aspects of a study including 4 phases.

##### Stages of Integration

All the studies enabled integration by connecting samples. The studies used either identical or nested sampling methods. On the basis of their designs and time orientation, they used samples sequentially or concurrently. Some studies (ie, [35,38,45,46]) made the integration at multiple points throughout their studies. They largely connected their samples, analyzed the data, merged their datasets, and interpreted their findings with an integrated view.

Some studies also enabled integration through the merging of both datasets in the data analysis stage. In an explanatory sequential design study on diabetes self-management [36], the themes from the qualitative analysis were merged with the demographic variables. In another study [33], descriptive measures of physical activity patterns and glucose levels of patients with type 2 diabetes were merged with the focus group data on the feasibility and acceptability of a new glucose monitoring and accelerometer technology.

The studies also presented various forms of data integration at the stage of interpretation or discussion. They identified themes or concepts and integrated their qualitative and quantitative findings into these themes [35,39,40,42,44,45]. In addition, the findings of a multiphase design study were reported in a series. After each stage was completed, its findings were written separately [46]. In some studies, specific findings were written in the separate parts of their reports and then an overall interpretation of the findings was made [34,38,43].

##### Prioritized Methods

More than half the studies evaluated their quantitative and qualitative methods equally; neither method was prioritized (8/14, 57%). Some studies with randomized controlled trial data [38,40,45] highlighted their comprehensive qualitative methodologies and their contributions to the results. For example, in order to completely measure young diabetic patients’ engagement with a new text messaging support system, a comprehensive content analysis of messages and message patterns of patients was performed [38]. Interestingly, in an explanatory design study [36], focus group results led researchers to reanalyze their quantitative data.

In another study [44], face-to-face interviews conducted before and after a text messaging trial provided important insights about the intervention’s technical features, tailored text messaging content, and motivational messaging. Researchers were guided to develop a better design for their apps. Three studies [34,35,43] primarily highlighted quantitative methods (QUAN + qual) and used qualitative methods to support quantitative data.

##### Data Collection Techniques

The most frequently used qualitative method in the studies was in-depth interviews (n=9). The studies conducted semistructured interviews [35,39,42,43,45,46], open-ended interviews [40], and motivational interviews [44]. Several studies conducted focus group meetings to collect data [33,36,37,41,42]. The interview or discussion duration varied from approximately 30 minutes to 90 minutes in these studies. In addition, 2 think aloud protocols [40,46], a content analysis [38], and a video-recording technique [46] were used to generate qualitative data.

Surveys were the most common quantitative data collection method (n=10), such as pre- and posttest questionnaires used to measure the usability of an interactive SMS text messaging intervention developed for patients with diabetes [40]. Perceptions of a group of American Indian women about gestational diabetes risk and related Internet and mHealth interventions were surveyed in a cross-sectional study [42]. In addition, there was a longitudinal cohort study [43] that collected survey data at multiple periods.

Quantitative data were also generated from descriptive measures, such as glucose and exercise levels of patients after a 72-hour trial of a continuous glucose monitoring system [33], usage data of patients after a 12-month trial of a text messaging support system [38], and interactive voice response calls and previous descriptive data [36,44].

##### The Methods of Legitimation and the Limitations Described

Although the studies in this review explicitly mentioned their procedures and strategies to increase the objectivity and credibility of their research, they did not use formal mixed-methods terminology. Therefore, the information on legitimation methods was extrapolated from the methodology and discussion sections of the studies.

Almost all the studies in this review applied an “inside and outside” legitimation method (13/14, 93%). For example, Nundy et al [43] assigned a team of 5 experienced research investigators to make an initial transcript coding and then randomly assigned 2 reviewers to code each transcript independently. In addition to peer reviews, team members’ views were important means for legitimation. For instance, conducting separate analyses helped the researchers compare their coding results and increased the validity of their study [39].

In addition to inside-outside legitimation, different methods of legitimation were identified in the studies. For instance, the qualitative and quantitative methods were used to complement each other and to minimize weaknesses (weakness minimization method) in a small-scale usability test study [37]. A small number of questionnaires were supported by conducting 10 different focus groups to increase the legitimacy of the results. The use of conversion legitimation was observed in 4 studies [35,43,45,46]. In 1 study [41], sequential legitimation was applied to minimize possible sequential order effects on the results.

Because the studies used either identical or nested sampling methods, sample integration legitimation was another frequently observed method of legitimation. Using similar samples in each phase minimizes lack of representativeness in qualitative findings and increases overall generalizability of the study [32]. For example, Grindrod et al [41] used identical samples in their qualitative and quantitative phases and performed multiple measurements to increase the validity of their small-scale intervention design, which lacked control group measurements.

Although various legitimation methods were used, they were also the source of study limitations. Many of the limitations were related to insufficient sample sizes and possible generalizability problems associated with them (ie, [35,36,38,44,46]). Sample selection problems and possible sample biases were also pointed out [33,39,40,42,43,45]. The duration of intervention [37,39,41], fidelity to the study [42], and other interventional issues, such as incentives (ie, lending cell phones, paying call charges) [37], and technical problems [39] were further limitations.

## Discussion

### Principal Findings

This scoping review identified comprehensive mixed-methods applications in the growing field of mHealth-based diabetes management. Mixed methods research can be effectively used if there is a strong and appropriate design dictated by research questions [48,49]. Creswell and Tashakkori [50] suggest using at least one clearly defined mixed methods question. Although none of the studies defined a separate mixed-methods research question and a very few studies (4/14, 29%) used formal mixed-methods terminology, the studies showed promise in their uses of multiple approaches and techniques. In addition to the common uses of mixed methods (triangulation and complementarity), studies with advanced designs presented various forms of combining different methods and techniques in sequential development of their studies. These studies are good examples for the field of diabetes management via mHealth technologies in which complicated interventions and multiple measurements are often required.

This study confirms the prominent role of qualitative methods in mixed-methods research. Qualitative measures were very important for the evolution of the majority of the papers reviewed, and they strongly influenced study findings. Because of the growing need for interpreting complex longitudinal studies and interventions [51] and big and sophisticated data coming from Web-based and mobile technology usage statistics, the importance of qualitative methods seems to be increasing.

The studies in this review presented various forms of integration at the method level. Some studies enabled integration at multiple points, linking their data collection and analysis at each point in the research. This is also noteworthy for mixed-methods studies in which integration is generally made at the level of data interpretation or discussion [52].

In terms of validity or legitimation strategies, little variation existed among the studies. In addition to inside-outside legitimation, these advanced designs should have also used weakness minimization and conversion legitimation methods because they are very specific to mixed-methods research.

The major limitations of the studies included in this review were related to data collection processes. Surveys were the most common data collection method used in the studies, but (as opposed to experimental studies) descriptive studies do not show causality, as they have poor control over external factors and external validity problems due to standardized question types. In addition, almost all studies acknowledged sampling problems (13/14, 93%). Small sample sizes, sampling bias, and use of convenient sampling are important barriers to the generalizability of findings in any type of study.

When various data collection techniques are used together, researchers are able to provide rich analysis and interpretations. This study shows that in addition to the common qualitative data collection techniques (focus group discussions and semistructured interviews), multiple data collection techniques (including think aloud protocols, interviews, field notes, content analysis, and evaluation forms) are used.

It is also noteworthy that these studies failed to discuss the advantages gained by using mixed-methods approaches. The majority of the studies (13/14, 93%) failed to provide a methodological discussion concerning the inconsistency or consistency between quantitative and qualitative data. Thus, this study supports Brown et al [24], who indicated that mixed-methods studies still lack justifications for using this approach.

### Study Strengths and Limitations

This study is an early exploration into the scope of mixed-methods studies in the field of mHealth-based diabetes management. This paper itself provides a demonstration of how to classify, analyze, and evaluate mixed-methods research. Regardless of field of interest, we believe many researchers could benefit from the guidelines, criteria, and examples provided in this review.

Although the database search was quite comprehensive, it was limited to the papers published in scientific journals and written in English. To increase the consistency of the coding, some established frameworks were used to identify design types [20], integration methods [31], and legitimation methods [32]. Because related information was not always clearly reported in the studies, some decision rules were applied and the most relevant choices presented in the frameworks were adopted. Another limitation is related to the coding process. Although the design types were classified by 2 coders, and a high level of agreement between them was established, the rest of the coding was done by the first author. However, to increase the confirmability of our analysis, she took the role of a “devil's advocate” with respect to the results, checking and rechecking each coding and analysis throughout the study.

### Conclusions and Future Recommendations

This review provided important insights into the evaluation of mixed-methods studies focusing on diabetes management via mHealth technologies. The prominent role of qualitative methods in mixed-methods research and tailored measures in diabetes self-management studies was confirmed, and the importance of using multiple methods and techniques in this field was emphasized. Future studies could continue to use different qualitative approaches and try to integrate new tools and approaches that seem to be effective in health care settings, such as participatory video, photovoice, online communities, and chat and discussion groups [3].

Considering the opportunities provided by mixed methods, it is surprising to see such a small number of studies in this field. The lack of systematic approaches in establishing rigor in mixed-methods studies seems to be a major barrier to the adoption and recognition of this research by a large group of social researchers [20]. However, in the field of mHealth-based diabetes management both usability studies and behavioral change interventions require complex measurements, so researchers could utilize the advantages of mixed methods more frequently. Future research should focus on the ways to improve precision in measurements by incorporating experimental designs with properly selected, adequate number of participants and new and creative data collection techniques.

In the future, researchers should incorporate formal mixed-methods terminology, starting with defining a mixed-methods question separately at the beginning, aiming to answer this question, and discussing the value of using the methodology at the end. In this growing field of diabetes management, future reviews should systematically analyze rigor in mixed-methods studies and compare the results with those of single-method studies.

## Abbreviations

CINAHL: Cumulative Index to Nursing and Allied Health Literature
mHealth: mobile health
NIDDK: National Institute of Diabetes and Digestive and Kidney Diseases
SMS: short message service
TExT-MED: Trial to Examine Text Message–Based mHealth in Emergency Department Patients with Diabetes
